# Some Experience with Cocaine Hydrochloride on the Eye

**Published:** 1885-03

**Authors:** B. H. Grove

**Affiliations:** Assistant Surgeon to the Buffalo Eye and Ear Infirmary


### Clinical Reports.

### Some Experience with Cocaine Hydrochloride on the Eye.

By B. H. Grove, M. D.,

Assistant Surgeon to the Buffalo Eye and Ear Infirmary.

The expression cocaine hydrochloride is used because that is preferred by a large majority of the authorities on chemical terminology. Cocaine was first used in this country to produce anæsthesia of the eye on Nov. 8, 1884. In from two to three weeks thereafter most of our medical journals were "charged and primed" with descriptions of what cocaine powder would do. It is not my purpose, therefore, to make a general review of this agent. I wish, however, to add to the galaxy of contributions already furnished a concise account of my experience with this agent on the eye. Continued experience with this drug must surely result beneficially, either by corroborating the investigations of others, or showing, in various ways, what it will not do. Up to the present time this agent seems especially applicable in ophthalmic surgery. With certain exceptions it causes complete anæsthesia of the conjunctiva and cornea, as well as deeper structures of the visual organ, but in no respect interferes with the vitality of the parts. Owing to such peculiar qualities this drug became, in a few days, one of especial merit. It was brought out from among the dust and rubbish of the materia medica storehouse, and was made important and conspicuous on account of its innate virtues. Up to Dec. 10, 1884, I had applied cocaine more than one hundred times to normal or diseased eyes. I wish to mention, especially, its mydriatic and anæsthetic properties. My experiments were made upon my own eyes, upon the eyes of rabbits, and patients attending the Buffalo Eye and Ear Infirmary. The cocaine was always obtained in powder form, as manufactured by Merck of Damstadt, and fresh solutions were made as required, water being the solvent. Such solutions remain clear for about two weeks, when microscopic growths appear. I have used one, two and four per cent. solutions. According to Dr. Squibb, these are only three-fourths as strong as the figures represent, owing to impurities of the alkaloid. On different days there was instilled in one of my eyes one drop of a two per cent. and four per cent. solution respectively. In each case my pupils began to dilate in sixteen minutes. The two per cent. solution caused no unpleasant sensation. Some dilatation was apparent fourteen hours afterwards. The accommodation was not affected. In the course of eight minutes partial anæsthesia of the cornea occurred. The four per cent. solution caused some smarting in the conjunctiva, and also a gummy, sticky feeling. Some dilatation remained twenty-four hours after its use. My accommodation was noticeably affected, since my near point had receded two inches. This endured for forty-five minutes. In most of my experiments I used a two per cent. solution. After the instillation of one drop, a slightly unpleasant sensation was said to be experienced in the conjunctiva. The same mucous membrane became slightly pale. The cornea became partially insensitive in from eight to fifteen minutes and continued so for about ten minutes. The pupil began to dilate in four to twenty minutes.

It is a curious fact, to which I have seen no reference made, that in dark brown irides the pupil never began to dilate before ten minutes, and in most cases not before sixteen minutes. In thirty cases there was only one exception. For this peculiarity I can assign no cause. The dilatation, at the end of about two hours, began to diminish. The accommodation was not affected, nor was there any apparent increase of tension of eyeball. I found a one per cent. solution caused dilatation about as rapidly and as extensively as a four per cent. solution. With this exception a four per cent. solution was in all respects more decided in its action. That the cocaine is absorbed, and passes through the structure of the cornea, can be nicely demonstrated by experimenting with rabbits. At various times I used two per cent. and four per cent. solutions. As a rule, there was dilatation of the pupil in from four to fifteen minutes. In one case, after several applications, no dilatation occurred. After dilatation was manifest, I drew off the aqueous humor by a hypodermic syringe, or else after thoroughly drying the conjunctival sac made paracentesis of the cornea, and drew up the escaped fluid of the anterior chamber into an ordinary eye-dropper. A few drops of this fluid, dropped in the eyes of other rabbits, caused dilatation in about ten minutes. To produce anæsthesia, for operation or otherwise, a two per cent. or four per cent. solution can be used. In case a two per cent. solution is used, the applications must be more frequent, and there is liable to be a loss of the solution from overflowing the lids. On the eye, therefore, a four per cent solution is the best. I proceed as follows: Ask the patient to look down, and let two drops fall upon the orbital conjunctiva. Repeat this proceedure every five minutes until four applications have been made. Then wait five minutes and begin the operation. If during the operation pain is experienced, apply the solution as becomes necessary. I have thus far, in my experience, with a few exceptions, been able to produce complete or nearly complete anæsthesia. I have made use of cocaine in one case of senile cataract extraction, various cases of iridectomy, pterygium strabismus; also, one case of sclerotomy and the removal of several foreign bodies from the cornea, besides some minor operations.

It is well known how essential perfect quietness is, in the operation for cataract extraction. The thoughtful operator was ever wont to thus question himself: Has the patient sufficient self control or should chloroform or ether be given, and the risk of vomiting be incurred? The decision is not always easily made. In this uncertainty cocaine comes to our aid. Its use is especially applicable in extraction cases, for with it, all reflex contractions of the globe are overcome, and thus the chances of the escape of vitreous are lessened—that complication which represents both the Scylla and Charybdis between which every operator on the eye must pass. In my case of cataract extraction, the various steps of the operation were made in their usual order, successfully. I mention this because in such a case the anæsthetic virtues of cocaine aı e well tested. After making the usual incision through the sclero-corneal border, and just before I drew out the iris to make iridectomy, I dropped two drops of the cocaine solution into the wound. This virtually amounts to the same thing as applying the anæsthetic to the anterior chamber by syringe, as some recent contributors have made especial mention of. After the operation the patient said she had experienced no pain whatever with the exception of that produced by the lower blade of the speculum pressing upon the infraorbital ridge. In fact, in all cases where the speculum is necessarily used I find the stretching apart of the eyelids is the cause of most of the pain. One of my cases of strabismus was divergent. Wishing to bring the optic axes for the far point parallel if possible, without making advancement of the internal rectus muscle, it was necessary to loosen the attachment of the external rectus and the capsule of tenon very thoroughly. The operation was made without any pain. In cases of strabismus, cocaine is a very serviceable anæsthetic, for with its employment one is able to judge accurately whether or no the tenotomy has been made sufficiently. With chloroform, or ether, one does not know until the effects have passed off. Other operations were made as a rule without pain. But it is in those cases of foreign bodies in the cornea where cocaine proves of service, especially to the general practitioner as well as the specialist. To remove these little foreign particles nicely, two factors are essential—a quiet eye and a steady hand. These being present, one person can accomplish about as much as another.

One week before I had obtained any cocaine, a young man came to my office with a particle of iron in the cornea of his left eye. He kept his eye in continual motion, but after some time, and causing much pain, I succeeded in removing the particle. Two weeks afterwards he returned with a piece of iron in the same cornea, only more deeply imbedded. After instilling a few drops of a four per cent. solution of cocaine, the particle was removed without causing any pain whatever. Simple blepharospasm of the lids and photophobia arising from various lesions of the cornea in a majority of cases can be overcome at once, after two or more instillations of a four per cent. solution. In some cases, however, complete anæsthesia of the cornea may be produced and still the photophobia remain as decided as before. I have likewise applied the cocaine before treating granulated lids with the copper crystal. For a time no pain was experienced, but in fifteen to twenty minutes it seemed to be more severe and last longer than when cocaine had not previously been used. My experience teaches that sometimes cocaine fails to be a perfect anæsthetic, even when fresh solutions are used. In one case of pterygium, after the use of a four per cent. solution for a number of times, only incomplete anæsthesia could be produced.

I will mention, also, a case of partial pannus of the cornea, where I wished to make an artificial pupil. Two drops of a fresh four per cent. solution were applied every five minutes for six times, still the anæsthesia of the conjunctiva and cornea was only partial. In many cases of lachrymal trouble I have injected cocaine solutions into the sac before passing Bowman's probes.

In most cases only partial anæthesia was produced. Although the most constant property of cocaine is its mydriatic effects, still there are cases where little, if any, dilatation can be produced. I will mention one case of hyperæsthesia of the retina. The cornea was clear but the pupil somewhat contracted. A four per cent. solution was used. The irides were dark brown. After thirty minutes the pupil had only slightly dilated, but not sufficiently for ophthalmoscopic examination. Twenty-four hours afterwards, when the effect of the cocaine had completely passed off, I found that one drop of a one-tenth of one per cent. solution of atropia sulphate dilated the same pupil in twenty minutes fully three times as much as the cocaine had produced in thirty minutes. Pardon me for referring here briefly to atropia as a mydriatic. We are all familiar with its peculiar action upon the iris, but the extreme sensitiveness of its nervous structure to this drug cannot be fully appreciated until very weak solutions are used. I made a solution of atropia sulphate so that one drop represented $\frac{1}{230,000}$ of a grain of the drug; one drop of that solution dilated the pupil in one hour and five minutes. In other words, put one grain of sulphate of atropia in nearly four gallons of water; mix well. One drop placed in the eye will cause dilatation of the pupil in about an hour. In such weak solutions, you understand, the accommodation is not at all affected and the dilatation disappears in a short time. Cocaine has been used hypodermically to produce anæsthesia for operations on the lids and enucleation of the eyeball. Most of the attempts were not altogether satisfactory, and in some cases when six drops of a four per cent. solution were injected, unpleasant symptoms were produced. The summary of my experience with cocaine on the eye is as follows:

1. That solutions continue clear for about two weeks, when a fungus appears. Such solutions retain their mydriatic properties but lose some of their anæsthetic virtues.

2. That one drop of a one per cent. solution dilates the pupil about as rapidly and as extensively as the same quantity of a four per cent. solution, but never causes any paralysis of accommodation.

3. That its mydriatic properties are noticed two or three times sooner in blue eyes than in dark brown.

4. That it will serve to dilate the pupil in most cases for ophthalmoscopic examination, but is not so reliable as sulphate of atropia, nor is the dilatation so extensive; therefore, when it is desired to examine the periphery of the lens or fundus of the eyeball, atropia is the better mydriatic.

For operations, a four per cent. solution is the best.

